# The role of the ER stress-response protein PERK in rhodopsin retinitis pigmentosa

**DOI:** 10.1093/hmg/ddx370

**Published:** 2017-09-27

**Authors:** Dimitra Athanasiou, Monica Aguila, James Bellingham, Naheed Kanuga, Peter Adamson, Michael E Cheetham

**Affiliations:** 1 UCL Institute of Ophthalmology, London EC1V 9EL, UK; 2 Ophthiris Discovery Performance Unit, GlaxoSmithKline Ophthalmology, Stevenage SG1 2NY, UK

## Abstract

Mutations in rhodopsin, the light-sensitive protein of rod cells, are the most common cause of dominant retinitis pigmentosa (RP), a type of inherited blindness caused by the dysfunction and death of photoreceptor cells. The P23H mutation, the most frequent single cause of RP in the USA, causes rhodopsin misfolding and induction of the unfolded protein response (UPR), an adaptive ER stress response and signalling network that aims to enhance the folding and degradation of misfolded proteins to restore proteostasis. Prolonged UPR activation, and in particular the PERK branch, can reduce protein synthesis and initiate cell death through induction of pro-apoptotic pathways. Here, we investigated the effect of pharmacological PERK inhibition on retinal disease process in the P23H-1 transgenic rat model of retinal degeneration. PERK inhibition with GSK2606414A led to an inhibition of eIF2α phosphorylation, which correlated with reduced ERG function and decreased photoreceptor survival at both high and low doses of PERK inhibitor. Additionally, PERK inhibition increased the incidence of inclusion formation in cultured cells overexpressing P23H rod opsin, and increased rhodopsin aggregation in the P23H-1 rat retina, suggesting enhanced P23H misfolding and aggregation. In contrast, treatment of P23H-1 rats with an inhibitor of eIF2α phosphatase, salubrinal, led to improved photoreceptor survival. Collectively, these data suggest the activation of PERK is part of a protective response to mutant rhodopsin that ultimately limits photoreceptor cell death.

## Introduction

Retinitis pigmentosa (RP) is a group of inherited diseases that cause the progressive dysfunction and degeneration of rod and cone photoreceptor cells in the retina leading to blindness. There are currently no effective treatments for RP. Inherited mutations in rhodopsin, the light sensing protein of rod cells, are the single most common cause of autosomal dominant RP, which primarily affect rod cells followed by the secondary loss of cone cells (1). The majority of mutations are class II mutations and cause protein misfolding, such as P23H, the most common mutation in the USA (2). The misfolded protein is retained in the endoplasmic reticulum (ER) and degraded by the proteasome. If mutant rhodopsin is not efficiently degraded it can aggregate and form intracellular inclusions, similar to those found in many other neurodegenerative diseases (3,4). P23H rhodopsin expression induces the unfolded protein response (UPR) (5), a signalling network that senses imbalances between protein synthesis, quality control and degradation in the ER.

The UPR has multiple mechanisms to restore proteostasis by reducing protein synthesis and upregulating factors that reduce misfolded proteins in the ER; for example, molecular chaperones to stimulate folding and ER-associated degradation (ERAD) factors to enhance degradation. The UPR has three different signalling proteins which are kept in an inactive state by binding to the ER-chaperone binding immunoglobulin protein (BiP, HSPA5); the inositol-requiring protein-1 (IRE-1), protein kinase RNA-like ER kinase (PERK), and activating transcription factor 6 (ATF6) (6–8). The PERK branch of the UPR mainly regulates protein synthesis. Upon ER stress, PERK oligomerises via its cytoplasmic domain, autophosphorylates and leads to phosphorylation of the eukaryotic initiation factor 2α (eIF2α), which activates the selective translation of activating transcription factor 4 (ATF4) and transcription of other UPR genes such as C/EBP homologous protein (CHOP), but also reduces protein synthesis. Protein synthesis is restored when the ATF4 downstream target and specific phosphatase, GADD34 dephosphorylates eIF2α. Upon failure of proteostasis restoration, the prolonged activation of UPR can initiate apoptosis (6,8). Indeed, increased levels of phosphorylated PERK (p-PERK) and phosphorylated eIF2α (p-eIF2α) have been found in several neurodegenerative diseases and models, such as Alzheimer’s disease (AD) (9), Parkinson’s disease (10), amyotrophic lateral disease (ALS) (11) and prion diseases (12) directing future studies on manipulation of the PERK branch. Suppression of p-eIF2α by genetically deleting PERK in AD mice restored levels of synaptic proteins and protected against synaptic and memory dysfunction (13). Furthermore, overexpression of GADD34 reduced eIF2α phosphorylation and increased neuronal survival in prion-disease mice (12). Moreover, treatment with a selective inhibitor of PERK, GSK2606414A, increased the survival of prion disease mice by reducing neuronal cell death (14). This PERK inhibitor was also protective in a *Drosophila* model of ALS (15).

The involvement of ER stress and activation of UPR in retinal degeneration has been examined by several studies [for reviews (16,17)]. For instance, UPR related genes such as BiP and the proapoptotic factor CHOP were found to be altered in P23H transgenic rats, suggesting that rod opsin misfolding might cause persistent ER stress that cannot be overcome (5). Additionally, overexpression of BiP reduced ER stress and protected against retinal degeneration in a P23H rat model (18), and ER stress was also observed in hT17M rhodopsin mice (19). Interestingly, other forms of retinal degeneration, not classically associated with ER protein misfolding, have also been reported to induce the UPR (20,21). We have recently shown that the use of drugs that intervene at different stages of proteostasis imbalance can be used as a therapeutic strategy to treat RP. More specifically, treatment with an Hsp90 inhibitor that induces heat shock protein expression improved retinal function and survival in P23H transgenic rats (22). Moreover, we showed that all the three UPR branches were enhanced by arimoclomol treatment, which also enhances the heat shock response, and this correlated with protection of photoreceptors in rhodopsin RP models (23).

Therefore, the role of ER stress in the pathogenesis of rhodopsin retinal degeneration is unresolved. For example, genetic ablation of CHOP does not protect against rhodopsin RP (24–26), whereas genetic depletion of ATF4 has been reported to protect against T17M mutant rhodopsin (27). Here we used a specific pharmacological inhibitor of PERK to probe the role of PERK activation in the pathogenesis of P23H related RP. The data show that PERK inhibition further impairs photoreceptor function and survival, while it enhances P23H rod opsin misfolding and aggregation. Furthermore, potentiation of eIF2α phosphorylation with salubrinal delayed photoreceptor degeneration. These data suggest that PERK is part of a protective cellular response to mutant rhodopsin and is not a good target for this form of rhodopsin RP.

## Results

The P23H-1 transgenic rat shows PERK activation (5) and undergoes fast retinal degeneration with 25% photoreceptor loss by postnatal day 15 (P15) compared with wild-type (WT) Sprague Dawley (SD) rats, which provides a rapid and robust model for the assessment of the ability of drugs to affect photoreceptor survival (22,23,28,29). GSK2606414A is a potent and selective inhibitor of PERK, it is functionally active in cells, and inhibits PERK with a selectivity of >385 fold over other eIF2α kinases and a panel of 294 kinases, and has excellent central nervous system (CNS) penetration following oral dosing (14,30). Therefore, we tested the ability of GSK2606414A (PERKi) to modify photoreceptor degeneration in the P23H-1 transgenic rat.

### PERK inhibition reduces visual function in P23H-1 rats

Rats were orally treated daily with a dose of 100 mg/kg PERKi, or vehicle, as this exceeds the dosing predicted to maximally inhibit PERK in the brain (14). Specifically, a single oral dose of 100 mg/kg/day PERKi in the rat yielded an area under the curve (AUC) exposure of 339,000 ng.h/ml (14 days dosing), whereas 50 mg/kg/day orally twice a day (BID) in mouse yielded an AUC of 13,913 ng.h/ml (5 weeks dosing), which was a fully efficacious dosing regimen in mouse neuroprotection studies (14). Treatment was initiated at three weeks of age (P21). At this stage, there is already retinal degeneration and this corresponds to a post symptom-onset treatment. The total levels of rhodopsin are reduced in this model compared with control SD rat retina, reflecting the loss of photoreceptor cells (Supplementary Material, Fig. S1) (31). Rats were monitored daily for body weight gain and for any adverse effects until the end of the treatment at P35. Rats tolerated the high dose of PERKi since no adverse effects were observed throughout the duration of treatment. As expected, and previously reported (14), PERKi led to a reduction in the age-related weight gain over the treatment period in the treated rats (Supplementary Material, Fig. S2A).

**Figure 1.**
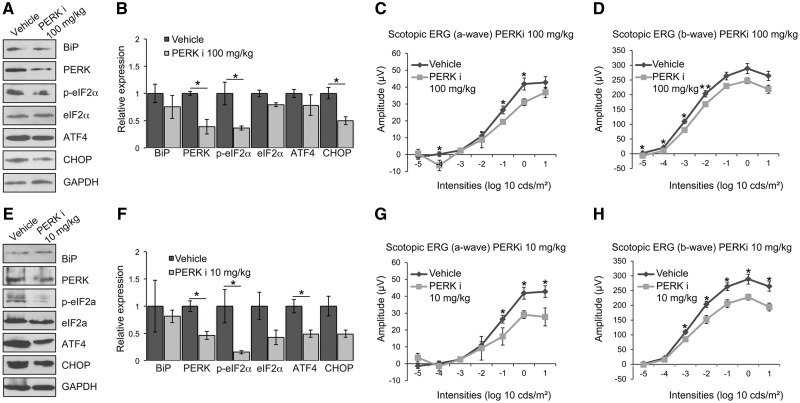
PERK inhibition reduces visual responses in P23H-1 rats. P23H-1 rats were treated from P21-P35 with either GSK2606414A (PERKi) or vehicle. (**A, E**) Representative western blot of retina lysates of P36 P23H-1 rats treated with 100 mg/kg (A) or 10 mg/kg (E) PERKi or vehicle for BiP, PERK, p-eIF2α, eIF2α, ATF4 and CHOP. GAPDH was used as a loading control. (**B, F**) Quantification of expression levels of BiP, PERK, p-eIF2α, total eIF2α, ATF4 and CHOP in P23H-1 rats after treatment with 100 mg/kg (B) or 10 mg/kg (F) PERKi. Densitometric analysis was used to calculate the levels of these proteins relative to vehicle; values are mean ± SEM *n* ≥ 4. (**C, D**) Scotopic ERG a-wave (C) and b-wave (D) amplitude results of P23H-1 rats (P36) treated from P21-P35 with either 100 mg/kg PERKi (*n = *8) or vehicle (*n = *6). (**G, H**) Scotopic ERG a-wave (G) and b-wave (H) amplitude results of P23H-1 rats (P36) treated from P21-P35 with either 10 mg/kg PERKi (*n = *8) or vehicle (*n = *6). Values are mean ± SEM, *P < 0.5, **P < 0.01, Student's *t* test.

Retinal lysates of vehicle and PERKi treated P23H-1 rats were analysed by immunoblotting to assess whether oral administration with PERKi successfully inhibited PERK and PERK-mediated downstream signalling. The expression of mutant P23H rhodopsin in P23H-1 rat retina leads to the induction of the UPR (5,23). Similarly, we observed increased levels of BiP, and p-eIF2α in P23H-1 vehicle retina compared with SD retina (data not shown). Treatment with PERKi significantly reduced the phosphorylation of eIF2α and the level of PERK and CHOP in P23H-1 retina, whereas the reduction in ATF4 did not reach significance (Fig. 1A and B). These data confirm access of PERKi to the rat retina, reduced PERK activity and inhibition of the PERK branch of the UPR.

Photoreceptor function was assessed by a full field scotopic electroretinogram (ERG) in dark-adapted rats at P35. Increasing light intensity correlates with a greater response of photoreceptor hyperpolarisation (a-wave), followed by the propagation of the signal through the retina with subsequent depolarisation (b-wave). Treatment with PERKi led to lower ERG responses as both a-wave and b-wave response amplitudes were reduced compared with vehicle-treated animals (Fig. 1C and D). This suggests that PERK inhibition impairs the visual function of P23H-1 rats.

T17M rhodopsin transgenic mice with genetically reduced levels of ATF4 were recently shown to have delayed photoreceptor degeneration (27). Therefore, it is possible that a partial inhibition of PERK signalling could have different consequences for photoreceptor survival from maximal inhibition. Treatment with PERKi at 10 mg/kg leads to a lower brain exposure to the inhibitor and a partial neuroprotective response in mice (14); therefore, we investigated if this lower dose of PERKi would also affect visual function. The lower dose of PERKi was also well tolerated and had a smaller effect on inhibiting body-weight gain compared with 100 mg/kg dose, suggesting a lower level of systemic PERK inhibition (Supplementary Material, Fig. S2B). The low dose treatment significantly reduced phosphorylation of eIF2α and the level of PERK and ATF4 in P23H-1 retina (Fig. 1E and F), whereas the reduction in CHOP level did not reach significance. These data suggest that the low dose of PERKi was also effective at inhibiting the PERK branch of the UPR in the rat retina. The low dose treatment also reduced both the a-wave and b-wave ERG responses of P23H-1 rats across a range of light stimuli (Fig. 1G and H), showing that a lower dose of treatment also affected visual function.

To investigate if the inhibition of PERK affected the other branches of the UPR in the retina, the retinal lysates from the high and low dose treatments were immunoblotted for IRE1 and phospho-IRE1 (p-IRE1) and ATF6. The data revealed no significant changes in IRE1 level or phosphorylation (Supplementary Material, Fig. S3), and no significant change in the level of cleaved ATF6 (Supplementary Material, Fig. S4). These data suggest that inhibition of PERK in the P23H-1 rat retina does not lead to a compensatory enhanced activation of either IRE1 or ATF6.

### PERK inhibition leads to enhanced photoreceptor cell death in P23H-1 rats

**Figure 2.**
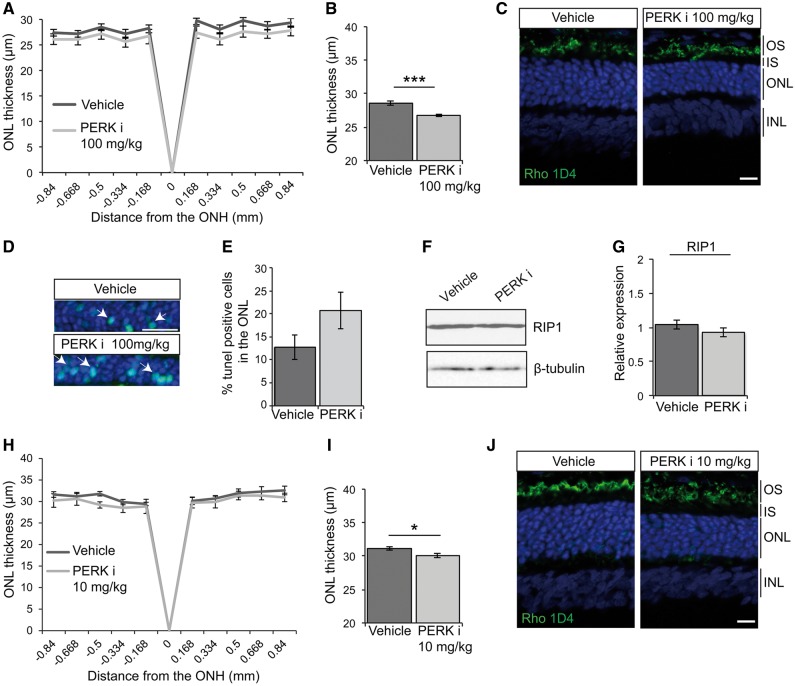
PERK inhibition reduces photoreceptor survival in P23H-1 rats. P23H-1 rats were treated from P21-P35 with either 100 mg/kg (**A–G**) or 10 mg/kg (**H–J**) PERKi or vehicle. (A, H) Spider plot showing P23H-1 ONL thickness at P36 after PERKi (*n = *6) or vehicle (*n = *6) assessed by OCT. (B, I) Mean ONL thickness across the whole retina (E). Values are mean ± SEM. *P < 0.05, ***P < 0.001, Student’s *t*-test. (C, J) Representative images of the retina from P23H-1 (P36) rats treated with vehicle or PERKi. Cryosections were stained with anti-rhodopsin antibody 1D4 (green), and DAPI (blue) as indicated. (D, E) TUNEL staining in the ONL of P23H-1 rats treated with 100 mg/kg PERKi or vehicle treated. Arrows highlight positive TUNEL cells. Scale bar 10 µm. (D) Percentage of TUNEL positive cells in the ONL quantified by scoring *n = *10 images each from 2 vehicle-treated rats and 2 PERKi-treated rats (100 mg/kg). Immunoblotting (F) and quantification (G) relative to vehicle of RIP1 immunoreactivity in PERKi (100mg/kg) and vehicle treated retinal lysates, β-tubulin was used as a loading control. Scale bar 20 µm.

To investigate whether the reduction in visual function correlated with a reduction in photoreceptor numbers, optical coherence tomography (OCT) was used to measure the thickness of the outer nuclear layer (ONL), as a marker of photoreceptor survival. The measurement of the ONL along the temporal-nasal meridian is shown in Figure 2A. Treatment with 100 mg/kg PERKi led to a significant reduction in ONL thickness across the whole retina (Fig. 2B), suggesting enhanced photoreceptor cell death. To confirm this observation, the eyes of vehicle and PERKi treated rats were analysed by histology (Fig. 2C). The DAPI stained ONL of PERKi treated retina was noticeably thinner than vehicle-treated P23H-1 retina.

Terminal deoxynucleotidyl transferase dUTP nick end labelling (TUNEL) was used to detect DNA fragmentation and measure apoptosis in the vehicle and PERKi treated P23H-1 retina. As expected with the active cell death associated with P23H expression, there was a high level of TUNEL reactivity in the vehicle-treated retina, which was further increased on PERKi treatment (Fig. 2D and E). Activated caspase 3 was not detectable by immunoblotting (Supplementary Material, Fig. S5), in agreement with published studies of the P23H-1 retina (32). In contrast, rod cell death by necroptosis has been reported in the P23H-1 retina with increased levels of receptor-interacting serine/threonine-protein kinase 1 (RIP1) (33). Furthermore, it has been suggested that GSK2606414A can also inhibit RIP1 (34). Therefore, the level of RIP1 in vehicle and high dose PERKi treated P23H-1 retina was investigated; the data show that there was no significant change in the level of RIP1 (Fig. 2F and G). This suggests that treatment with PERKi does not have a major effect on RIP1 in the rat retina.

Analyses of the 10 mg/kg treated animals by OCT and histology (Fig. 2H–J), confirmed that the ONL was also significantly reduced at the lower dose of PERKi. Collectively, these data show that PERK inhibition compromises visual function and accelerates photoreceptor loss in the P23H-1 rat.

### Prolonging eIF2α phosphorylation enhances P23H-1 photoreceptor survival

**Figure 3.**
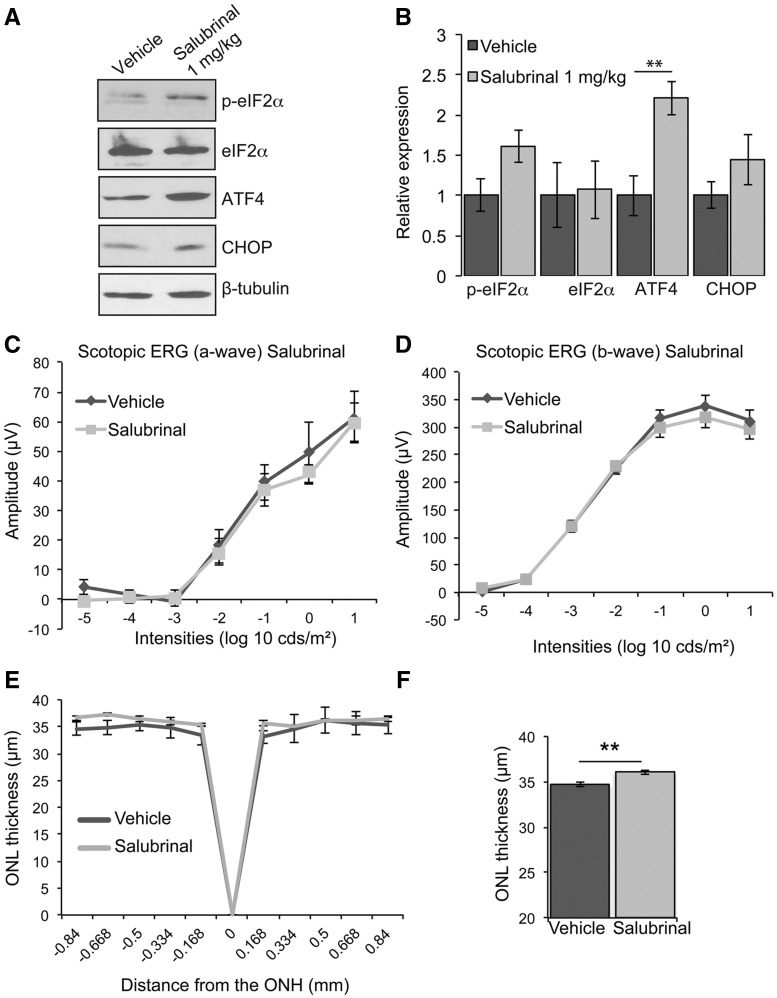
Salubrinal enhances photoreceptor survival in P23H-1 rats. P23H-1 rats were treated from P21-P35 with either 1 mg/kg salubrinal or vehicle. (**A**) Representative western blot of retina lysates of P23H-1 rats treated with salubrinal or vehicle for p-eIF2α, eIF2α, ATF4, and CHOP. β-tubulin was used as a loading control. (**B**) Quantification of expression levels of p-eIF2α, total eIF2α, PERK ATF4 and CHOP relative to vehicle. (**C**, **D**) Scotopic ERG a-wave (C) and b-wave (D) amplitude results of P23H-1 rats (P36) treated from P21-P35 with either salubrinal (*n = *8) or vehicle (*n = *9). (**E**) Spider plot showing P23H-1 ONL thickness at P36 after salubrinal (*n = *5) or vehicle (*n = *4) assessed by OCT. (**F**) Mean ONL thickness across the whole retina Values are mean ± SEM, **P < 0.01, Student’s *t*-test.

As inhibition of PERK activity was detrimental to P23H expressing photoreceptors, we wanted to test the hypothesis that prolonging PERK signalling could be protective. Salubrinal is a specific small molecule inhibitor of eIF2α dephosphorylation (12), and inhibiting the activity of eIF2α phosphatases can potentiate PERK signalling. Therefore, we treated P23H-1 rats daily with 1 mg/kg of salubrinal, a dose that has previously been shown to affect eIF2α phosphorylation in the brain and retina (12,35). Treatment with salubrinal led to a significant increase in the level of ATF4, and increased p-eIF2α and CHOP, although this did not reach statistical significance (Fig. 3A and B). There was no effect of salubrinal on visual function as determined by ERG (Fig. 3C and D); however, there was a small, but significant, increase in ONL thickness as determined by OCT (Fig. 3E and F), suggesting that salubrinal can enhance photoreceptor survival in the P23H-1 rat.

### PERK inhibition enhances P23H rod opsin aggregation

**Figure 4.**
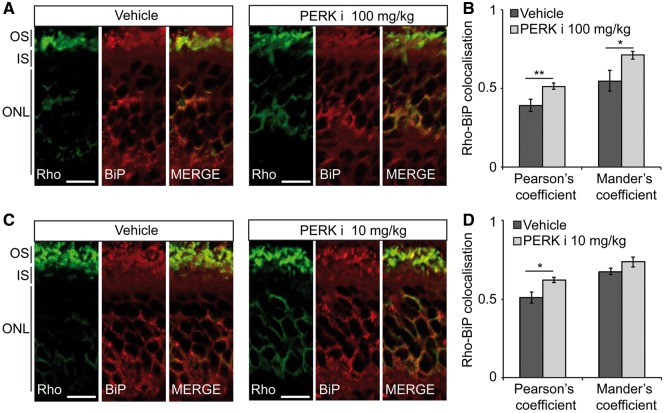
PERK inhibition increases rhodopsin ER localisation in P23H-1 rats. P23H-1 rats were treated from P21-P35 with either (**A, B**) 100 mg/kg or (**C, D**) 10 mg/kg PERKi or vehicle. (A**,** C) Immunohistochemistry of rhodopsin (green) and BiP (red) immunoreactivity in the ONL and inner segment (IS). Scale bar 10 µm. (B, D) Rhodopsin-BiP co-localisation quantified by calculating the Pearson's and Mander's co-localisation coefficients using the JaCOP plug-in and ImageJ software, *n* = 18 images each from 3 vehicle-treated mice and 3 PERKi-treated mice. Values are means ± SEM, *P < 0.05, ***P < 0.001, unpaired two-sided Student's *t* test.

To investigate the potential mechanisms underlying the accelerated retinal degeneration following PERK inhibition, immunohistochemistry was used to investigate rhodopsin traffic. Treatment with either high or low dose PERKi led to an increase in the co-localisation of rhodopsin with BiP in the ER as determined by co-localisation analyses (Fig. 4). This suggests that there is potentially increased rhodopsin ER retention, or reduced rhodopsin ERAD.

**Figure 5.**
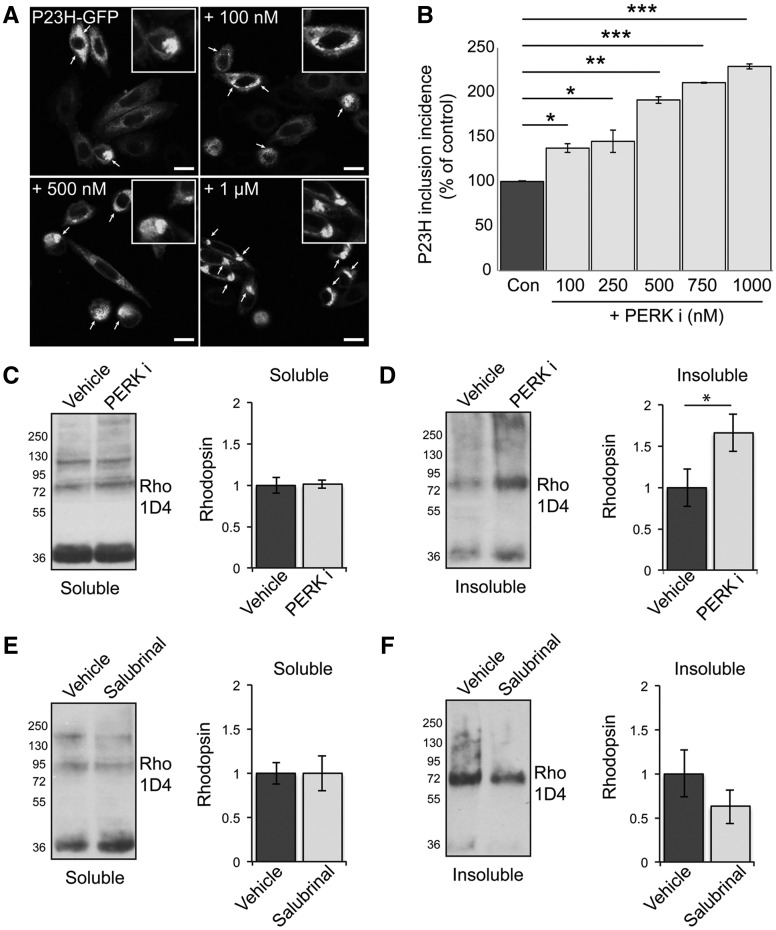
PERK inhibition increases P23H rhodopsin aggregation. **(A, B)** SK-N-SH cells were transfected with P23H-GFP rod opsin. Three hours post-transfection and after 2 h recovery in serum cells were either left untreated or treated with 100, 250, 500, 750 mM and 1μM of PERKi for 18 h prior to fixation. (A) Representative confocal images of P23H-GFP untreated rod opsin or treated with 100mM, 500 mM and 1 μM PERKi as indicated. Scale bar 10 μm. Magnified cell images are shown in insets. (Β) The incidence of inclusion formation of P23H-GFP in the absence and presence of PERKi at the indicated concentrations was assessed by scoring the percentage of cells with rod opsin P23H-GFP inclusions in 8 fields of ∼100 transfected cells. (**C–F**) Retinae of P23H-1 rats treated from P21-P35 with either 100 mg/kg PERKi (C,D), or salubrinal (E, F) or vehicle were analysed by a sedimentation assay. Fractions were immunoblotted with the 1D4 antibody against rhodopsin. Densitometric analysis was used to calculate the levels of soluble rhodopsin (C,E) relative to the vehicle treated and insoluble rhodopsin (D,F) relative to the vehicle after normalisation to soluble rhodopsin. Values are means ± SEM, *n* ≥ 4 (biological replicates). Error bars represent standard error, *P < 0.5, **P < 0.01, ***P < 0.001 Students *t*-test.

P23H rhodopsin can aggregate and form intracellular inclusions on heterologous expression in cell culture models (36). Therefore, we investigated any potential direct effects of PERK inhibition on rod opsin folding, traffic and aggregation in cell culture and used the incidence of intracellular inclusions as a surrogate marker of protein misfolding and aggregation (23,36). Treatment with a range of concentrations of PERKi led to a dose dependent increase in mutant rod opsin inclusions (Fig. 5A and B).

To investigate if PERK inhibition altered rhodopsin aggregation *in vivo*, a differential sedimentation assay (22,23,28) was used to assess the amount of soluble and insoluble rhodopsin present in P23H-1 retina after manipulation of eIF2α phosphorylation. Interestingly, treatment with PERKi did not lead to a difference in the amount of soluble rhodopsin (Fig. 5C), suggesting that there was not a major change in rhodopsin degradation following PERK inhibition. In contrast, there was a significant increase in the amount of insoluble rhodopsin following PERK inhibition (Fig. 5D). Treatment with salubrinal did not affect soluble rhodopsin levels, but led to a small non-significant decrease of insoluble rhodopsin (Fig. 5E and F). These data suggest that PERK activation can reduce mutant rod opsin aggregation and its inhibition exacerbates mutant rhodopsin ER retention, misfolding and aggregation.

## Discussion

The UPR is an adaptive response that aims to reduce ER stress through reducing demand (protein translation); increase ER exit (producing more factors to enable folding); and remove misfolded proteins through ERAD; however, sustained activation can have deleterious effects and lead to cell death. Although the role of ER stress and activation of the UPR in neurodegeneration has been extensively investigated, its role in disease progression remains controversial. The retina offers an excellent paradigm to study the role of cell stress in neurodegeneration, due to its accessibility, well-characterised function and the detailed understanding of genetic causes of retinal degeneration. Furthermore, it can be targeted with systemic, or local (intravitreal) drug delivery, or gene therapy. In this study, we used GSK2606414A, a selective inhibitor of PERK (30), to further investigate the role of this branch of the UPR in retinal degeneration associated with misfolded P23H rhodopsin. Our data are consistent with PERK activation having a protective role against P23H at these relatively early stages of disease.

We used the well-characterised P23H-1 transgenic rat model, which has well documented UPR induction (5,23). This model has 9 copies of a 15 kb genomic fragment containing the mouse rhodopsin gene with P23H mutated. At one month of age the P23H allele represents approximately 43% of all the rhodopsin mRNA in hemizygous rats with the remainder being driven by the two WT rhodopsin alleles; therefore, this is an overexpression model expressing almost double the level of rhodopsin mRNA than a WT rat (31). In contrast, the heterozygous P23H knock-in mouse, which has a single P23H allele and expresses P23H mRNA at a level equivalent to one WT allele, appears to have a different profile of ER stress with lower levels of IRE-1 activation and negligible PERK activation (25,37). Therefore, the activation of PERK in the P23H rat might be related to the high levels of expression of the P23H transgene on a background of two copies of WT rat rhodopsin. This finding questions the potential direct role for PERK activation in mediating photoreceptor cell death at the correct gene dosage. A directly pro-apoptotic role of PERK also appears unlikely, as CHOP ablation does not appear to affect P23H mediated photoreceptor cell death (24,25). The genetic reduction of ATF4, however, protects against T17M rhodopsin, suggesting a role for the PERK downstream target ATF4 in rhodopsin RP cell death (27). This difference could be due to developmental adaptation to reduced ATF4 levels leading to an increase in unknown protective factors, or alternatively different mechanisms of disease for T17M compared with P23H rhodopsin. However, the loss of CHOP does not protect the T17M model either (26), suggesting that any beneficial effects of ATF4 reduction are not mediated through reducing CHOP. We used a dose of PERKi that had been shown to provide only partial neuroprotection in the mouse to test if lower levels of PERK inactivation and reduced ATF4 could protect the P23H-1 rat. However, rats show a greater exposure to PERKi on an equivalent dose basis than mouse and therefore, the level of PERK inhibition in the retina, even at this low dose, may have been too high to test if partial inhibition could be protective. Nevertheless, our data provide the first direct evidence that inhibiting PERK activity is detrimental in P23H mediated RP. The use of a chemical inhibitor also circumvents any problems of developmental changes that can confound genetic targeting approaches that are used to study this adaptive response.

Recently, it was suggested that GSK2606414A, and the related compound GSK2656157, might not be specific to PERK, but can also inhibit RIP1 (34). Furthermore, it was recently shown in the P23H-1 rat model that rod photoreceptors die by necroptosis via an RIP1/RIP3/DRP1 mechanism, and that rod cell death could be reduced by treatment with the RIP1 inhibitor necrostatin 1 s (Nec1s) (33). We investigated whether treatment with PERKi affected RIP1 and we did not observe any change in RIP1 levels after PERKi treatment, whereas treatment with Nec1s has been shown to lead to a strong reduction in RIP1 level (33). Therefore, if PERKi was mainly acting to inhibit RIP1 in the retina, it should have a protective effect on rod cell survival and greater effect on RIP1, which we did not observe. Collectively, these data further suggest that it is the activity against PERK that is leading to the enhanced cell death following PERKi treatment.

Interestingly, prolonging eIF2α phosphorylation with salubrinal had a mild protective effect on the P23H transgenic rat retina. It was recently reported that salubrinal can reduce TUNEL reactivity in P23H transgenic mice and P23H knock-in mice without any WT allele (P23H/-), which both have PERK activation (35). It is not clear at the moment why the one copy of the knock-in allele on a null background (P23H/-) would lead to greater PERK activation than the knock-in allele on a WT background (P23H/+), but it could relate to the formation and traffic of proteins to the specialised ciliary outer segment. P23H mutant rhodopsin alone cannot mediate the formation of an outer segment in the absence of any WT allele (38,39), and disruptions in cilia/outer segment traffic have been reported to induce the UPR. For example, the loss of BBS12 leads to activation of PERK and treatment of BBS12 knock-out mice with guanabenz, an inhibitor of eIF2α dephosphorylation, valproic acid and caspase 12 inhibitor led to enhanced photoreceptor survival (40). Therefore, potentiating PERK activity appears to be protective in P23H and ciliopathy models, where PERK is activated. In the future, it will be interesting to test if enhancing PERK activity is protective in the absence of any PERK activation. It is also possible, however, that at later stages of disease prolonged PERK activation might become detrimental through globally inhibiting translation, but at the peak of photoreceptor cell death, the main role appears to be beneficial.

The potential mechanisms of how PERK inhibition might affect photoreceptor viability were investigated and revealed that PERK inhibition led to an increase in rhodopsin aggregation in both cells and the P23H-1 rat retina. We also observed increased co-localisation of rhodopsin with the ER marker BiP, suggesting enhanced ER retention or increased expression. Previously, we have observed that increased PERK activation in the P23H-1 rat, stimulated by the heat shock protein coinducer arimoclomol, correlated with a decrease in photoreceptor cell death and reduced rhodopsin aggregation (23). It is possible that PERK activation can have dual effects on mutant rhodopsin expression; to reduce protein synthesis, which lowers the burden of translating mutant rhodopsin; and to increase the expression of protective factors, such as BiP, EDEM1, ERdj5 and VCP/p97 that can reduce mutant rhodopsin aggregation and stimulate ERAD (41–44). The combination of these effects leads to lower mutant rhodopsin expression, less ER accumulation and aggregation.

In *Drosophila melanogaster*, moderate ER stress caused by the loss of the fly rhodopsin specific chaperone, NinaA, protected photoreceptors from cell death (45) and another study showed that stimulating ERAD protects against mutant rhodopsin (46). Similarly, our data support that stimulation rather than inhibition of the UPR is potentially more beneficial to this type of rhodopsin RP. This might not be the case for other types of rhodopsin RP or other forms of retinal degeneration, where induction of the UPR is a secondary consequence of the disease process, such as those associated with TDP43 alteration in splicing (15), or PrP aggregation (12,14). Nonetheless, it appears that in diseases such as P23H-1 rhodopsin RP, where protein misfolding in the ER is part of the primary disease mechanism, the UPR plays an important role as the first line of defence against proteotoxic cell stress.

## Materials and Methods

### Materials

P23H rod opsin-GFP plasmid was as previously described (4). The mouse monoclonal primary antibody 1D4 (mAb; 1: 1000) against rod opsin was a gift from Professor Robert Molday (Department of Biochemistry and Molecular Biology, University of British Columbia, Canada). Protease inhibitor cocktail (PIC), phosphatase inhibitor cocktail (PhIC), 4’, 6-diamidino-2-phenylindole dihydrochloride (DAPI) and staurosporine were from Merck-Sigma Hertfordshire, UK) PERK rabbit monoclonal (mAb; 1: 1000), eIF2α rabbit polyclonal antibodies (pAb; 1: 1000) and cleaved caspase 3 (Asp175) rabbit polyclonal antibodies (pAb; 1: 1000) were from New England Biolabs (Hitchin, UK). BiP rabbit polyclonal antibody (pAb; 1: 3000), RIP1 rabbit polyclonal (pAb; 1: 1000), β-tubulin mouse monoclonal antibody (mAb; 1: 2000) and GAPDH mouse monoclonal antibodies (mAb; 1: 40000) were from Merck-Sigma. ATF6 rabbit polyclonal (pAb; 1: 250) and ATF4 rabbit polyclonal (pAb; 1: 250) were from Santa Cruz (California, USA). CHOP (GADD113) mouse monoclonal (mAb; 1: 500) was from Proteintech (Manchester, UK). Phosphorylated eIF2α (pSer51) (E1F2S1) rabbit polyclonal (pAb; 1: 500) and IRE1α rabbit polyclonal (pAb; 1: 1000) were from Abcam (Cambridge, UK). Phosphorylated IRE1α (pSer724) rabbit polyclonal (pAb; 1: 1000) was from Thermo Fisher Scientific (Massachusetts, USA). Goat anti-rabbit and anti-mouse secondary antibodies conjugated to horseradish peroxidase were from Pierce (Cramlington, UK). Goat anti-mouse Alexa Fluor 488 (1: 1000) and goat anti-rabbit Alex Fluor 594 (1: 1000) secondary antibodies conjugated IgGs (1: 1000) were from Invitrogen (Paisley, UK). The eIF2a inhibitor Salubrinal (#324895), the luminata forte and luminata Crescendo HRP substrates were purchased from Millipore (UK) Limited. Lipofectamine and Plus reagents were purchased from Thermo Fisher Scientific. *In situ* cell death detection kit (Fluorescein) was from Sigma (UK).

### Animals

All procedures were conducted according to the Home Office (UK) regulations, under the Animals (Scientific Procedures) Act of 1986, and with the approval of local UCL-Institute of Ophthalmology, London, UK ethics committee. The P23H-1 line rats were kindly provided by Professor Matt LaVail, (University of California, San Francisco, USA) and crossed with WT Sprague Dawley (SD) rats to generate hemizygous P23H-1 rats. SD rats were purchased from Harlan (Blackthorn, UK). All animals were housed under a 12: 12 light dark cycle, with food and water available *ad libitum*.

### GSK2606414A and salubrinal treatment

P23H-1 rats were orally gavaged once daily from P21 to P35 with 10 mg/kg and 100 mg/kg GSK2606414A or vehicle at a volume of 0.2 ml per 50 g of rat or with 1 mg/kg Salubrinal dissolved in DMSO and then in milk and vehicle (milk). Rats were monitored daily for any adverse effects and for body weight gain.

### Electroretinography (ERG)

At P35, rats were dark-adapted overnight in a ventilated light-tight box and anaesthetized with Ketamine/Xylazine at 0.2 ml/100 g intraperitoneally (i.p.). Full-field scotopic ERG was carried out using the Diagnosys system and Espion software (Diagnosys, Lowell, MA) under red-light conditions and as previously described (28). Briefly, simultaneous bilateral recordings were taken using scotopic ERG protocols. Flash stimuli (10 ms to 1 ms duration, repetition rate 0.2 Hz) were presented via an LED stimulator (log intensity -5 to + 1) under scotopic conditions. ERG responses were collected with the Espion software for analysis. Statistical analysis was performed using Student's *t* test.

### Optical coherence tomography (OCT)

Rat retinae were imaged using the Bioptigen Spectral-domain ophthalmic imaging system (SDOIS) as previously described (28). Briefly, image acquisition was obtained by using the rectangular scanning protocol consisting of a 2 mm by 2 mm perimeter with 750 A-scans (lines) and 5 B-scans (frames) with a 60 Frames/B-scan. The Bioptigen InVivoVue Diver 2.0 was used to enable manual segmentation of the retinal layers and the outer nuclear layer thickness was measured after exporting results from Diver to Excel. Statistical analysis was performed using Student's *t* test.

### SDS-PAGE and western blot

Retinae were extracted and lysed with ice-cold RIPA buffer (50 mM Tris-HCl pH 8, 150 mM NaCl, 1 mM EDTA, 1% NP-40, 0.1% SDS, 0.05% sodium deoxycholate) containing 2% (v/v) mammalian protease inhibitor cocktail (PIC) 2% (v/v) mammalian phosphatase inhibitor cocktail. Retina lysates were sonicated for 2 × 30 s, centrifuged for 15 min at 12 000× g and at 4 °C and diluted in 5× sodium dodecyl sulphate (SDS) sample loading [0.0625 M Tris pH 6.8, 2% (w/v) SDS, 30% (v/v) glycerol, 20% (v/v) β-mercaptoethanol] to a final concentration of 1×. Samples for rod opsin were not denatured whereas samples for all the other antibodies were heated at 95 °C for 5 min before they were resolved by SDS-polyacrylamide gel electrophoresis (SDS-PAGE) and western blot. Proteins were transferred to nitrocellulose with 25 mM Tris, 192 mM Glycine, 0.01% SDS (w/v) and 20% (v/v) Methanol. To prevent non-specific binding, membranes were blocked at 4 °C with either 5% (w/v) Marvel Milk in PBS with 0.1% (v/v) Tween buffer or and 5% (w/v) BSA in TBS with 0.1% (v/v) Tween buffer. Immunodetection of proteins of interest was carried out using the primary and secondary antibodies described in Materials section. Proteins were detected using the Luminata Forte and Luminata Crescendo HRP substrate kits. Rhodopsin sedimentation assay was performed as described previously (22,23,28).

### Immunohistochemistry

Eyes were dissected and fixed overnight in 4% paraformaldehyde at 4 °C following by cryoprotection with 30% sucrose in PBS. Eyes were then frozen and cryosectioned as described (22). Eye sections were incubated in blocking buffer [3% bovine serum albumin (BSA) and 10% normal goat serum in PBS] for 1 h at room temperature before incubation with primary antibodies, as indicated. DAPI staining was used for nuclei visualisation. Terminal deoxynucleotidyl transferase dUTP nick end labeling (TUNEL) assay was performed using an *in situ* cell death detection kit (Fluorescein) following the manufacturer’s instructions.

### Cell transfection, drug treatments and immunofluorescence

SK-N-SH human neuroblastoma cells were maintained and transfected essentially as previously described (41). Three hours after transfection and 2 h after recovery in serum, cells were either left untreated or treated with 100 nM to up to 1 μΜ GSK2606414A for 18 h. Immunofluorescence was performed as previously described (44).

### Image analysis

Image acquisition of cells and retina was obtained using Carl Zeiss LSM700 laser-scanning confocal microscope. Images were exported from Zen 2009 software and prepared using Adobe Photoshop and Illustrator CS4. Cell morphology studies were scored as previously described (36). The Pearson's and Mander's co-localisation co-efficients in cells co-stained using Rho-1D4 and anti-BiP antibodies were measured with JaCOP plug-in and ImageJ software.

## Supplementary Material

Supplementary Material is available at *HMG* online.

## Supplementary Material

Supplementary Data
