# Supplementary material for: The role of the ER stress-response protein PERK in rhodopsin retinitis pigmentosa
Source: Hum Mol Genet. 2017 Sep 27;26(24):4896–905. doi: 10.1093/hmg/ddx370 (PMC5868081; doi:10.1093/hmg/ddx370)
Supplement: Supplementary Data [file ddx370_supplementary_material.pdf]

Supplementary material
The role of the ER stress response protein PERK in rhodopsin retinitis pigmentosa
Athanasiou et al

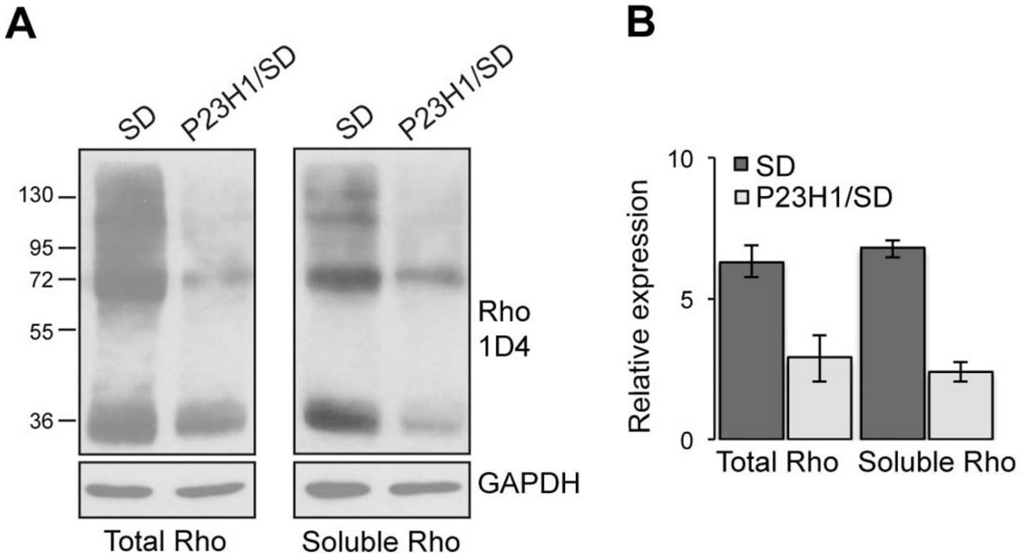

Figure S1. Rhodopsin expression levels in P23H-1 rat retina. (**A**) Immunoblot showing total and soluble rhodopsin levels in control SD rat retina and P23H-1 retina at P36. Retinal lysates were immunoblotted with the 1D4 antibody against rhodopsin. GAPDH was used as a loading control. The position of molecular weight markers is indicated on the left in kDa. (**B**) quantification of total and soluble rhodopsin levels. Densitometric analysis was used to calculate the levels of total and soluble rhodopsin. Values are mean ± SEM n≥3.

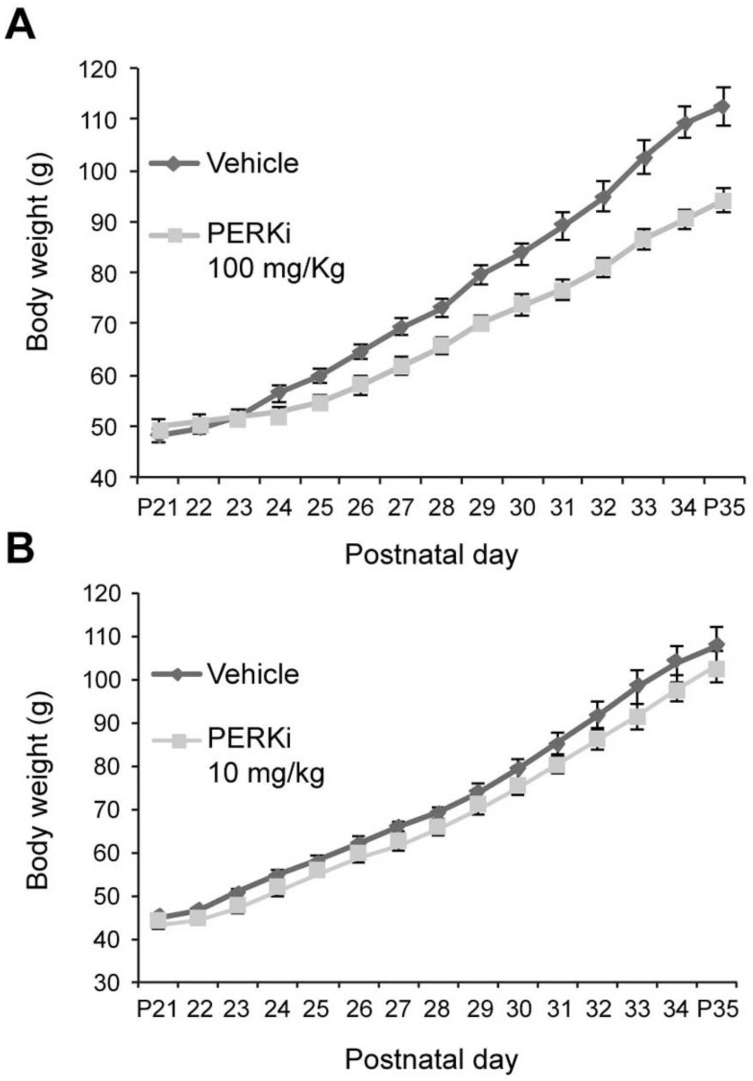

Figure S2. GSK2606414A reduces age-related body weight gain

P23H-1 rats were treated from P21-P35 with either (**A**) 100 mg/kg or (**B**) 10 mg/kg GSK2606414A (PERKi) or vehicle. GSK2606414A and vehicle were administered daily via oral gavage and rats were monitored daily for any adverse effects and for body-weight gain. (**A**) The high dose GSK2606414A-treated rats (n=8) had a lower increase in body weight as opposed to the vehicle-treated rats (n=6). (**B**) The low dose GSK2606414A-treated rats (n=8) also showed a reduced age-related increase in body weight compared to the vehicle-treated rats (n=6).

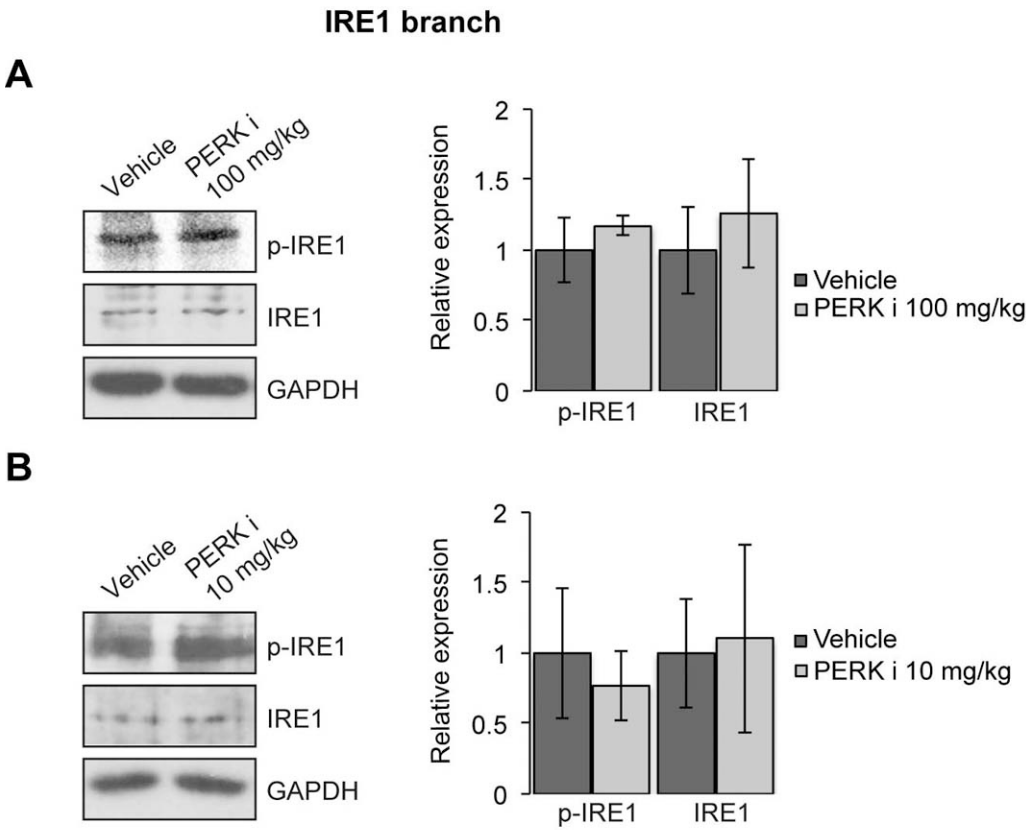

Figure S3. PERK inhibition does not alter IRE1 in P23H-1 rat retina

P23H-1 rats were treated from P21-P35 with either GSK2606414A (PERKi) or vehicle. (**A, B**) Representative western blot of retina lysates of P36 P23H-1 rats treated with 100 mg/kg (**A**) or 10 mg/kg (**B**) PERKi or vehicle for phosphor-IRE1 (p-IRE1) and IRE1. GAPDH was used as a loading control. Quantification of expression levels of p-IRE1 and IRE1 in P23H-1 rats after treatment with 100 mg/kg (**A**) or 10 mg/kg (**B**) PERKi. Densitometric analysis was used to calculate the levels of these proteins relative to vehicle; values are mean ± SEM n≥3.

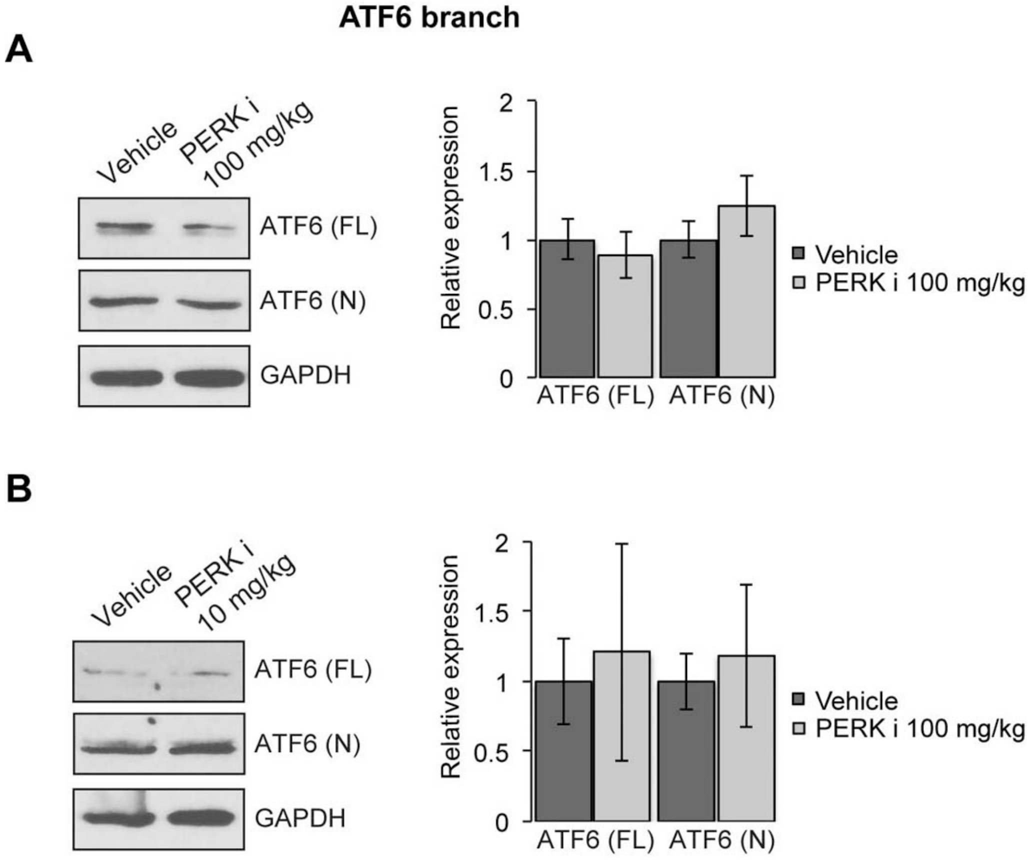

Figure S4. PERK inhibition does not alter ATF6 in P23H-1 rat retina
P23H-1 rats were treated from P21-P35 with either GSK2606414A (PERKi) or vehicle. (**A, B**) Representative western blot of retina lysates of P36 P23H-1 rats treated with 100 mg/kg (**A**) or 10 mg/kg (**B**) PERKi or vehicle for full length ATF6 (ATF6 (FL)) and cleaved activated ATF6 (ATF6 (N)). GAPDH was used as a loading control. Quantification of expression levels of ATF6 (FL) and ATF6 (N) in P23H-1 rats after treatment with 100 mg/kg (**A**) or 10 mg/kg (**B**) PERKi. Densitometric analysis was used to calculate the levels of these proteins relative to vehicle; values are mean ± SEM n≥3.

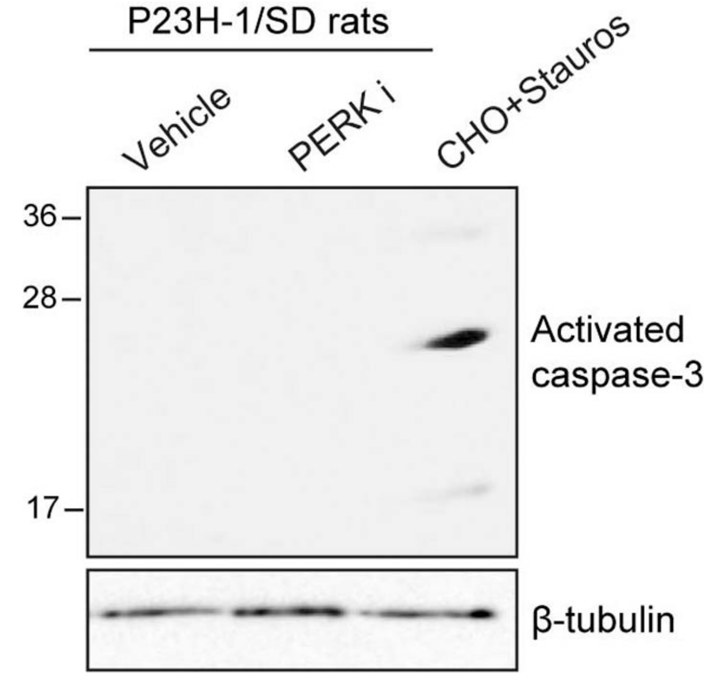

Figure S5. Lack of detectable activated caspase 3 in P23H-1 rat retina

P23H-1 rats were treated from P21-P35 with either GSK2606414A (PERKi) or vehicle. Representative immunoblot of retina lysates of P36 P23H-1 rats treated with 100 mg/kg PERKi or vehicle for activated caspase 3. CHO cells treated with staurosporine (Stauros), 1 μM for 18 hours, were used as a positive control. β-tubulin was used as a loading control.
